# Diabetic Impairment of C-Kit^+^ Bone Marrow Stem Cells Involves the Disorders of Inflammatory Factors, Cell Adhesion and Extracellular Matrix Molecules

**DOI:** 10.1371/journal.pone.0025543

**Published:** 2011-10-03

**Authors:** Tao-Sheng Li, Satoshi Ikeda, Masayuki Kubo, Mako Ohshima, Hiroshi Kurazumi, Yoshihiro Takemoto, Kazuhiro Ueda, Kimikazu Hamano

**Affiliations:** 1 Department of Stem Cell Biology, Nagasaki University Graduate School of Biomedical Science, Nagasaki, Japan; 2 Department of Surgery and Clinical Science, Yamaguchi University Graduate School of Medicine, Yamaguchi, Japan; Northwestern University, United States of America

## Abstract

Bone marrow stem cells from diabetes mellitus patients exhibit functional impairment, but the relative molecular mechanisms responsible for this impairment are poorly understood. We investigated the mechanisms responsible for diabetes-related functional impairment of bone marrow stem cells by extensively screening the expression levels of inflammatory factors, cell cycle regulating molecules, extracellular matrix molecules and adhesion molecules. Bone marrow cells were collected from type 2 diabetic (db/db) and healthy control (db/m+) mice, and c-kit^+^ stem cells were purified (purity>85%) for experiments. Compared with the healthy control mice, diabetic mice had significantly fewer c-kit^+^ stem cells, and these cells had a lower potency of endothelial differentiation; however, the production of the angiogenic growth factor VEGF did not differ between groups. A pathway-focused array showed that the c-kit^+^ stem cells from diabetic mice had up-regulated expression levels of many inflammatory factors, including *Tlr4*, *Cxcl9*, *Il9*, *Tgfb1*, *Il4*, and *Tnfsf5*, but no obvious change in the expression levels of cell cycle molecules. Interestingly, diabetes-related alterations of the extracellular matrix and adhesion molecules were varied; *Pecam*, *Mmp10*, *Lamc1*, *Itgb7*, *Mmp9*, and *Timp4* were up-regulated, but *Col11a1*, *Fn1*, *Admts2*, and *Itgav* were down-regulated. Some of these changes were also confirmed at the protein level by flow cytometry analysis. In conclusion, c-kit^+^ bone marrow stem cells from diabetic mice exhibited an extensive enhancement of inflammatory factors and disorders of the extracellular matrix and adhesion molecules. Further intervention studies are required to determine the precise role of each molecule in the diabetes-related functional impairment of c-kit^+^ bone marrow stem cells.

## Introduction

In the past decade, a number of studies have demonstrated that stem cells of bone marrow origin play very important roles in repairing/regenerating various organs [1]–[7], including the injured heart and vessels, through direct regeneration (cell differentiation/maturation) or indirect mechanisms (paracrine effects) [8]–[11]. Clinical trials have also attempted to treat ischemic heart diseases and peripheral arterial diseases by implanting autologous bone marrow-derived stem cells [12]–[17]. Some of these clinical trials have reported improvements in the clinical symptoms and regional perfusion of ischemia after treatment, but the therapeutic benefits observed were very marginal and mild, especially in patients of advanced age and those with diabetes and other diseases.

The inadequate efficiency of stem-cell-based therapy in patients with advanced age and other complications may be at least partially associated with the poor quality of cells used for implantation because aging and diabetes have previously been demonstrated to decrease the number and impair the function of bone marrow stem cells [18]–[22]. Therefore, attenuation of the impaired function of bone marrow stem cells should be a new approach to enhance the benefit of stem-cell-based therapy in these patients.

Our previous study found that oxidative stress likely contributes to the functional impairment of bone marrow stem cells in type 2 diabetic mice [23], but the precise mechanisms are not clearly understood. Using a pathway-focused microarray, we extensively compared the expression levels of inflammatory cytokines, cell cycle regulating factors, cell adhesion molecules and extracellular matrix molecules in c-kit^+^ bone marrow stem cells from diabetic mice and normal healthy mice, and we then attempted to further uncover the complex molecular mechanisms responsible for the diabetes-associated functional impairment of bone marrow stem cells.

## Materials and Methods

### Animals

We used 12-week-old male C57BLKS/J Iar-+Lep^db^/+Lep^db^ (db/db) mice (SLC, Japan); this mouse strain is characterized by the spontaneous development of type 2 diabetes mellitus (DM). C57BLKS/J Iar-m+/+Lep^db^ (db/m+) mice were used as healthy controls. We measured the body weight and blood glucose levels of all mice before sacrifice for the collection and isolation of bone marrow stem cells. All experiments were approved by were approved by the Institutional Animal Care and Use Committee of Yamaguchi University (#2006012), and animal procedures were performed in accordance with institutional and national guidelines.

### Collection, purification, and culture of c-kit^+^ bone marrow stem cells

Bone marrow cells were collected from the femur and tibia, and mononuclear cells were isolated by density gradient centrifugation. Stem cells expressing c-kit (c-kit^+^) were separated using a magnetic cell sorting system, as described previously [10], [19], [23]. Approximately 1.5% (0.86% to 2.12%) of the bone marrow mononuclear cells expressed c-kit, and the purity of sorted cells was approximately 90%.

Purified c-kit^+^ stem cells were seeded on 4-well chamber culture slides (Nalge Nunc International) coated with 10 µg/ml fibronectin (Invitrogen) at a density of 2×10^5^ cells/ml in RPMI 1640 medium supplemented with 10% fetal bovine serum (HyClone), 100 units/ml penicillin, and 100 µg/ml streptomycin (Gibco). Cells were incubated at 37°C in 5% CO_2_ [10], [19].

### ELISA

To measure the production of growth factors from c-kit^+^ stem cells, the supernatant was collected after 3 days of cell culture and stored at −80°C. The concentrations of VEGF, bFGF, and IL-1β in conditioned medium were measured with ELISA kits (R&D Systems), as described previously [10], [19].

### Immunocytochemistry

For immunostaining analysis of endothelial differentiation, the cells were fixed in 1% formaldehyde after 7 days of culture. After blocking with Protein Block Serum-free (Dako), cells were reacted with phycoerythrin-conjugated anti-mouse vascular endothelial (VE)-cadherin antibody (Santa Cruz). Nuclei were stained with 4,6-diamino-2-phenylindole dihydrochloride (DAPI). The numbers of positively stained cells were counted under fluorescence microscopy with 200-fold magnification. Twenty random microscopic fields were selected in each chamber, and the mean numbers of positively stained cells per field were used for statistical analysis [10], [19], [23].

### Gene expression analysis by pathway-focused GEArray

We compared the expression of inflammatory cytokines, cell cycle regulating factors, cell adhesion molecules and extracellular matrix molecules in c-kit^+^ bone marrow stem cells from diabetic and normal healthy mice using signaling pathway-specific gene expression GEArray systems (SuperArray Bioscience Corporation). The microarrays were used according to the manufacturer's instructions. Briefly, total RNA was extracted from freshly purified c-kit^+^ bone marrow stem cells. Using the reagents provided, cDNA was prepared from a mixture of purified total RNA of four independent samples by reverse transcription, biotinylated with Biotin-16-dUTP (Roche), and then hybridized under precisely specified conditions to a positively charged nylon membrane containing the arrayed DNA. The arrays were visualized using a chemiluminescent detection system (LAS-1000, FUJIFILM). Loading differences were accounted for on the basis of the intensity of the hybridization signals compared to the housekeeping genes, and then gene expression was quantified by scanning densitometry [24].

### Flow cytometry

To confirm the expression levels of genes observed using the pathway-focused GEArray at the protein level, we collected mononuclear cells from mice. Cells were blocked with 0.5% BSA for 10 min and labeled with FITC-conjugated anti-mouse c-kit^+^ antibody for 30 min. After washing, cells were then incubated with antibodies against TNFR2, TLR4, integrin-α_v_, PECAM-1, fibronectin, or laminin (BD Bioscience). Respective isotype controls were used as a negative control. Quantitative flow cytometry analysis was performed using a FACSCalibur (Becton Dickinson) [7], [21].

### Statistical analysis

All results are presented as the mean ± SD. Statistical significance between two groups was determined using the 2-tailed unpaired *t*-test (Dr. SPSS II, Chicago, IL). Differences were considered significant when p<0.05.

## Results

### Diabetes decreased the number and impaired the function of c-kit^+^ bone marrow stem cells

**Figure 1:**
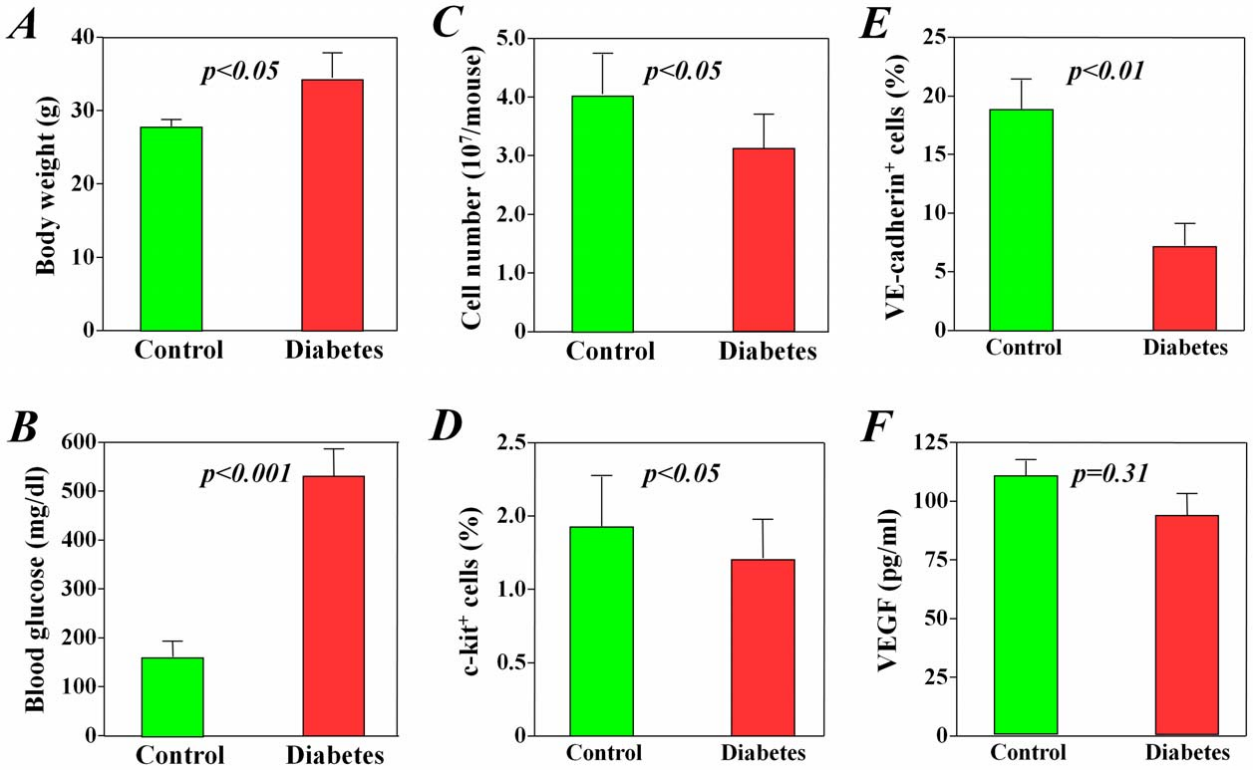
Differences in number and function of bone marrow stem cells between diabetic and healthy mice. Compared with healthy control mice, diabetic mice had significantly lower body weights (A) and higher blood glucose levels (B). Significantly fewer total bone marrow mononuclear cells were harvested from diabetic mice than from healthy control mice (C), and the subpopulation of c-kit+ stem cells was significantly decreased in diabetic mice (D). After 7 days of culture, the c-kit+ stem cells from diabetic mice showed significantly less differentiation into VE-cadherin-positive endothelial cells than did cells from healthy control mice (E), but the production of VEGF did not differ between the two groups (F).

When compared with healthy mice, the db/db diabetic mice had significantly higher body weights (*p<0.05*, *Figure 1A*) and blood glucose levels (*p<0.001*, *Figure 1B*), confirming that these mice had early-stage type 2 diabetes mellitus (body weight loss at late-stage in these obesity type 2 diabetes mice). Although all procedures of cell collection and isolation were performed in the same way, significantly fewer bone marrow mononuclear cells were collected from diabetic mice than from healthy mice (*p<0.05*, *Figure 1C*). Furthermore, the subpopulation of c-kit^+^ stem cells in freshly collected bone marrow mononuclear cells was also significantly smaller in diabetic mice than that from healthy control mice (*p<0.05*, *Figure 1D*).

After 7 days of culturing purified c-kit^+^ stem cells, we found that cells from diabetic mice had poorer attachment and expressed lower levels of VE-cadherin when compared with cells from healthy control mice (*p<0.05*, *Figure 1E*). However, the production of VEGF from c-kit^+^ stem cells did not differ significantly between groups at 3 days after culture (*p = 0.31*, *Figure 1F*), in agreement with the results of our recent study using bone marrow mononuclear cells from diabetic patients [25]. The production of bFGF and IL-1β was not detectable by ELISA.

### Diabetes increased the expression of inflammatory-related factors in c-kit^+^ bone marrow stem cells

**Figure 2:**
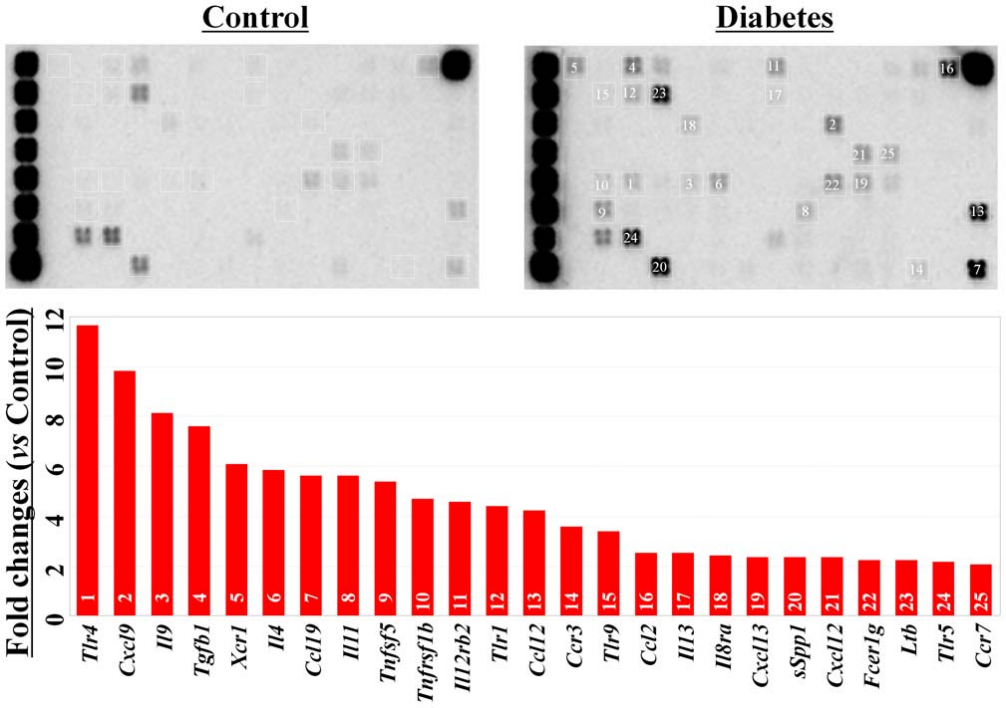
Pathway-focused GEArray analysis of the expression of inflammatory-related factors. Compared with the healthy control mice, the expression of inflammatory-related genes was extensively up-regulated in the c-kit+ bone marrow stem cells from diabetic mice.

We compared the expression levels of inflammatory-related factors in freshly purified c-kit^+^ stem cells between diabetic and healthy control mice. Among the 84 genes included in the pathway-focused GEArray system, 25 genes were up-regulated more than 2-fold in diabetic mice when compared with healthy control mice (*Figure 2*). These genes included *Tlr4*, *Cxcl9*, *Il9*, *Tgfb1*, *Il4*, *ccl19*, *Il11*, and *Tnfsf5*. No inflammatory-related gene was down-regulated more than 2-fold in the c-kit^+^ stem cells of diabetic mice.

**Figure 3:**
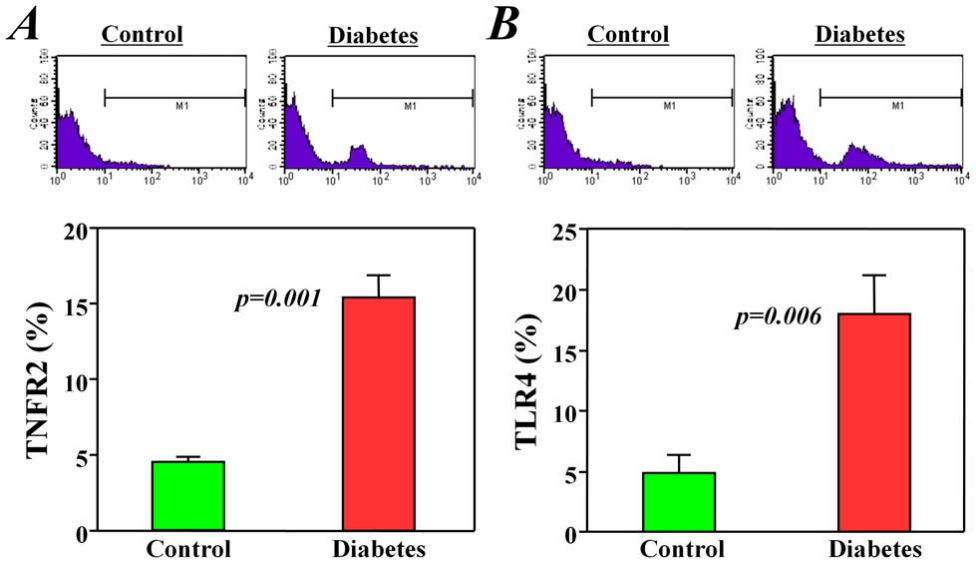
Expression of tumor necrosis factor receptor 2 (TNFR2) and toll-like receptor 4 (TLR4) in bone marrow stem cells. Flow cytometry analysis showed that the positive expression levels of TNFR2 and TLR4 in the c-kit+ bone marrow stem cells from diabetic mice were significantly higher (>3-fold) than in cells from healthy control mice.

Furthermore, we confirmed some of the GEArray data at the protein level using flow cytometry analysis. Only approximately 4.5% of c-kit^+^ stem cells from healthy control mice positively expressed TNF receptor 2, but the expression of this protein was observed in approximately 15% of c-kit^+^ stem cells from diabetic mice (*p<0.01*, *Figure 3A*). Similarly, the expression of toll-like receptor 4 was observed in approximately 20% of c-kit^+^ stem cells from diabetic mice but in only 5% of those from healthy control mice (*p<0.01*, *Figure 3B*).

### Diabetes did not change the cell cycle-related factors in c-kit^+^ bone marrow stem cells

**Figure 4:**
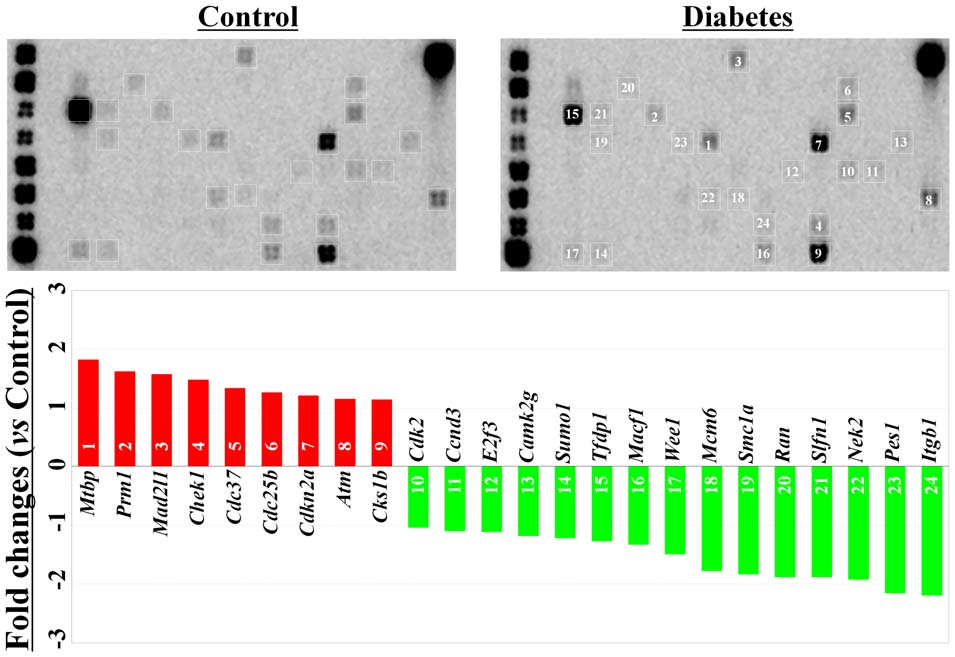
Pathway-focused GEArray analysis of cell cycle-related factors. The expression of cell cycle-related genes was clearly detected in the c-kit+ bone marrow stem cells from both diabetic mice and healthy control mice. However, the difference observed in the expression levels between groups was very mild (<2-fold).

The expression of cell cycle-related factors in c-kit^+^ stem cells was also compared between diabetic and healthy control mice. Although many of the cell cycle genes were clearly detected by the GEArray system (*Figure 4*), only two genes, *Pes1 and Itgb1*, were changed (down-regulation) more than 2-fold (2.2- and 2.3-fold, respectively) in diabetic mice when compared with healthy control mice (*Figure 4*). *Itgb1* is a well-known cell adhesion molecule, and we will discuss the data for this protein in detail below.

### Diabetes induced varying changes in the expression levels of ECM and adhesion molecules in c-kit^+^ bone marrow stem cells

**Figure 5:**
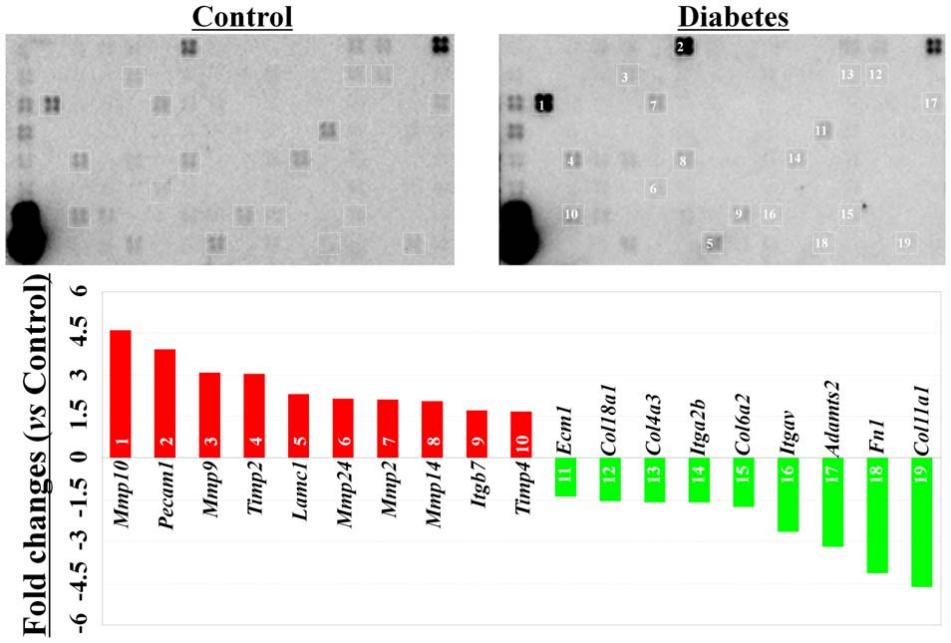
Pathway-focused GEArray analysis of the gene expression of extracellular matrix and adhesion molecules. Compared with the healthy control mice, 10 genes were up-regulated and 9 genes were down-regulated in the c-kit+ bone marrow stem cells from the diabetic mice.

Interestingly, diabetes induced varying changes in the expression of ECM and cell adhesion molecules in c-kit^+^ stem cells. Among the 84 genes included in the GEArray system, 10 genes (*MMP10*, *Pecam1*, *MMP9*, *Timp2*, *Lamc1*, *Mmp24*, *Mmp2*, *Mmp14*, *Itgb7*, and *Timp4*) were up-regulated, and 9 genes (*Col11a1*, *Fn1*, *Adamts2*, *Itgav*, *Col6a2*, *Itga2b*, *Col4a3*, *Col18a1*, and *Ecm1*) were down-regulated more than 1.5-fold in diabetic mice when compared with those from healthy controls (*Figure 5*).

**Figure 6:**
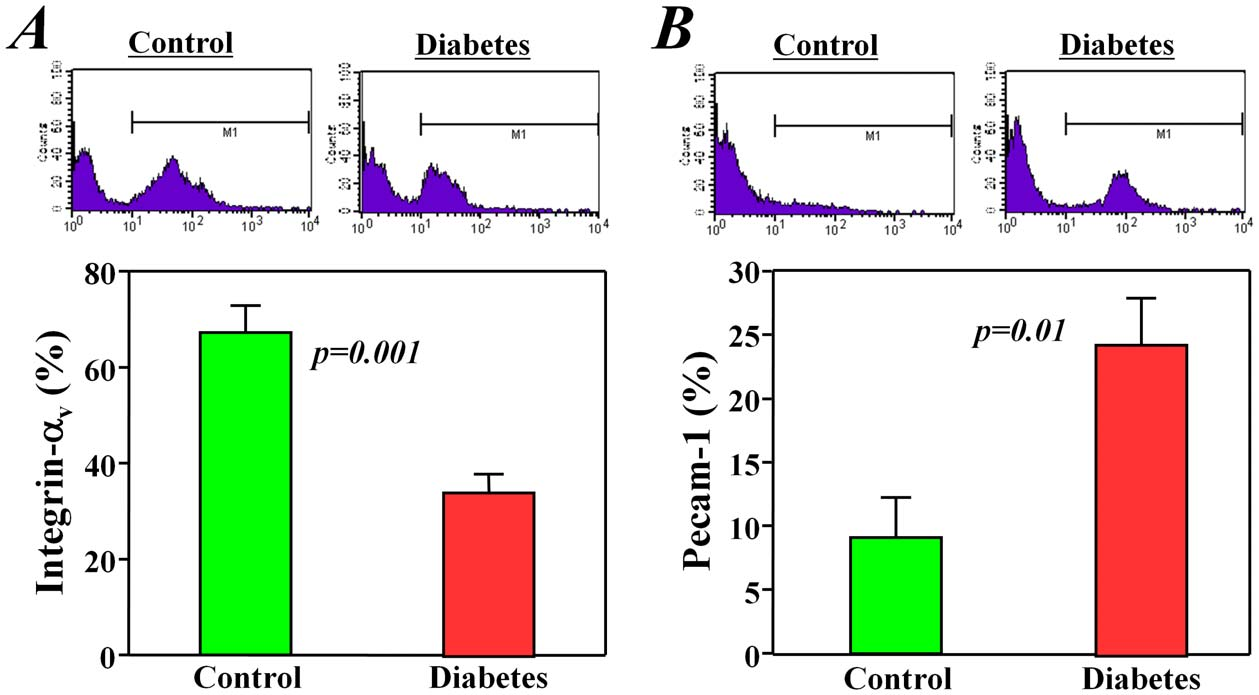
Expression of the adhesion molecules integrin-av and Pecam-1 in bone marrow stem cells. Flow cytometry analysis showed that the expression of integrin-av (A) was decreased but that of Pecam-1 (B) was increased in the c-kit+ bone marrow stem cells from diabetic mice when compared with those from healthy control mice.

Furthermore, we examined some of these changes at the protein level using flow cytometry analysis. We confirmed that the expression of one important cell adhesion molecule, integrin-α_v_, was significantly decreased (*p<0.01*, *Figure 6A*), but the expression of another important cell adhesion molecule, Pcam-1, was increased (*p<0.01*, *Figure 6B*) in c-kit^+^ stem cells from diabetic mice when compared with healthy controls.

**Figure 7:**
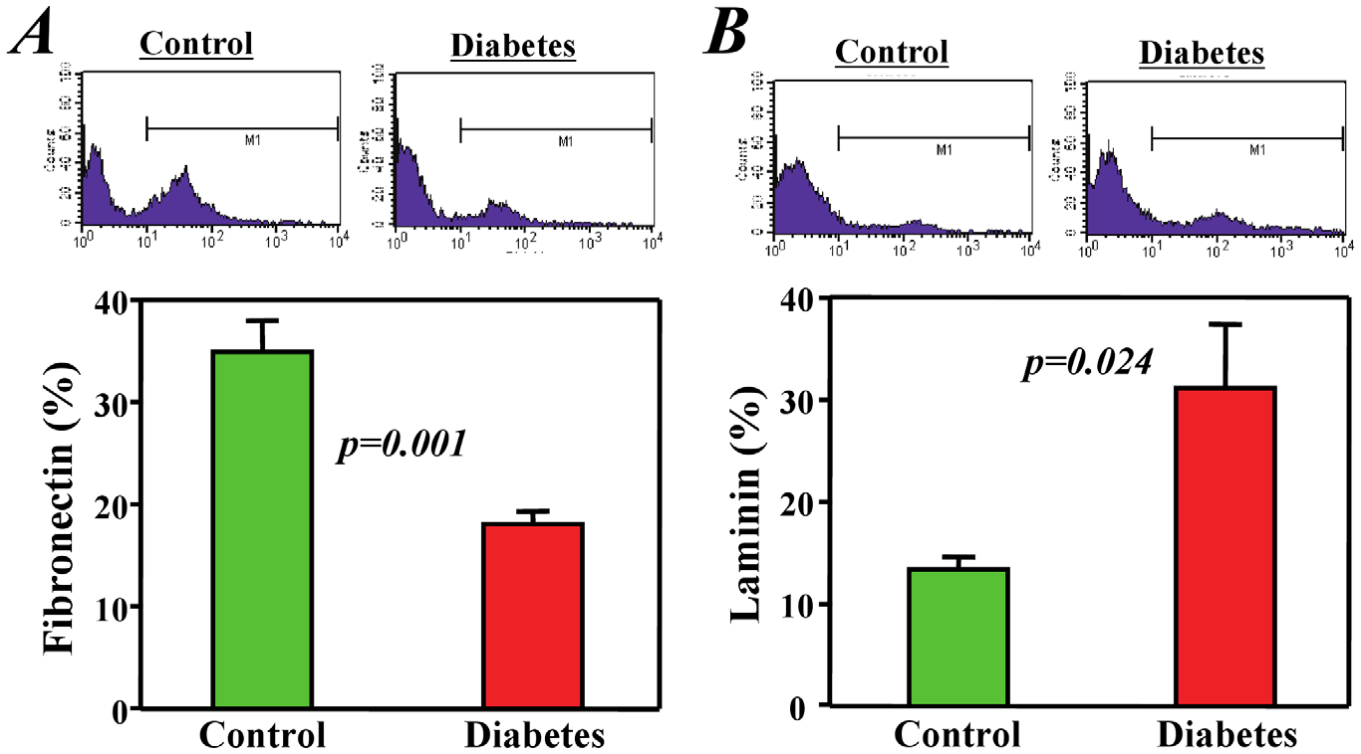
Expression of the extracellular matrix molecules fibronectin and laminin in bone marrow stem cells. Flow cytometry analysis showed that the expression of fibronectin (A) was decreased but that of laminin (B) was increased in the c-kit+ bone marrow stem cells from diabetic mice when compared with cells from healthy control mice.

Similarly, diabetes induced varying changes in ECM protein expression in c-kit^+^ bone marrow stem cells. One important ECM protein, fibronectin, was significantly decreased (*p<0.01*, *Figure 7A*), but laminin, another important ECM protein, was increased (*p<0.01*, *Figure 7B*) in c-kit^+^ stem cells from diabetic mice when compared with healthy controls.

## Discussion

Diabetes is well known to induce renal failure, impair hematopoiesis, and increase the risk of cardiovascular diseases [26], [27], but the precise mechanisms responsible for these diabetes-associated complications are not understood. In addition to hematogenesis, increased evidence has shown that bone marrow stem cells likely play important roles in repairing various tissues/organs [1]–[7]. Therefore, the reduced number and impaired function of bone marrow stem cells may contribute to the development of many complications. If we can understand the molecular mechanisms associated with the functional impairment of bone marrow stem cells, new approaches may be found to prevent and treat these diabetes-associated complications.

In this study, we purified c-kit^+^ bone marrow stem cells from early-stage diabetic mice (12 weeks old) for analysis and confirmed that diabetes is associated with a decrease in the number of c-kit^+^ bone marrow stem cells and that diabetes partially impairs their endothelial differentiation function [18]–[23].

Using a screening analysis of three of the most interesting pathways in this study, we obtained several interesting findings. First, the extensive enhancement of inflammatory factors in c-kit^+^ bone marrow stem cells from diabetic mice supported our speculation that systemic and local inflammatory microenvironments play central roles in the decrease in the number and functions of bone marrow cells. In fact, diabetes is considered an inflammatory disease that is characterized by increases in oxidative stress (e.g., the oxidation of glucose) and advanced glycation end-products (AGE) [23], [28], which induce the up-regulation of many inflammatory cytokines, such as MCP-1, TNF-α, IL-1, and IL-6 [29], [30]. We found that expressions of TNF receptor 2 and toll-like receptor 4 were significantly increased in these c-kit^+^ bone marrow stem cells from diabetic mice. Although it has reported that TNF receptor 2 is required for angiogenesis in response to ischemia [31], TNF receptors as mediators of apoptosis have been classically considered as negative regulators in the immune and hematopoietic systems to suppress hematopoietic stem and progenitor cell function [32]. Toll-like receptor 4 is primarily identified as an innate immune receptor, but it has been recently found to play a negative role in stem cells [33], [34]. Although further intervention experiments are required to identify the key mediators and the pathway signaling in detail, inflammatory stimulation likely contributes to the functional impairment of bone marrow stem cells in diabetes patients.

Because the number of c-kit^+^ bone marrow stem cells was decreased in diabetic mice, the proliferative activity of stem cells may be enhanced to maintain the homeostasis of the stem cell pool [35], [36]. However, the expression levels of cell cycle-related factors in c-kit^+^ bone marrow stem cells did not obviously change between diabetic and healthy control mice. This result may due to that fact that the cells used for analysis were harvested at the early-stage of diabetes, at which the number of stem cells in the bone marrow may not have dramatically decreased. Alternatively, the proliferative activity of bone marrow stem cells in diabetic mice may be suppressed by the inflammatory microenvironment or other unknown mechanisms.

The pattern of changes in the expression levels of ECM and adhesion molecules was quite interesting. As inflammation is known to change the expression of ECM and adhesion molecules in various types of cells, it is not difficult to conjecture that the systemic and local inflammatory microenvironment of diabetes may induce these changes in the expression levels of bone marrow stem cells. Inflammatory stimulation usually enhances the expression levels of ECM and adhesion molecules. However, approximately half of these ECM and adhesion molecules were down-regulated in bone marrow stem cells of diabetic mice. Thus, the mechanism responsible for the diabetes-induced disorder of ECM and adhesion molecules in bone marrow stem cells is not likely as simple as inflammatory stimulation. The down-regulation of *Itgb1* was detected in the stem cells of diabetic mice (Figure 4). This result agreed well with results of our previous study that functional impairment of *ex vivo* expanded bone marrow stem cells was related to decreased expression of integrin-β_1_ [37]. Although the ECM and adhesion molecules are generally known to be critical for cell growth, it is poorly understood on their precise role in regulating the growth and differentiation of stem cells. In this study, we found that the expression of integrin α_v_ was decreased in these c-kit^+^ bone marrow stem cells from diabetic mice. Agree well with our data, it has been demonstrated that oxidized low density lipoprotein induce functional impair of endothelial progenitor cell by down-regulation of E-selectin and integrin α_v_β_5_ [38]. However, it has reported that the α_v_ subunit of integrin does not appear to be involved with the attachment of embryonic stem cells to fibronectin [39]. An interventional approach of inflammatory inhibition will help us to demonstrate the causal relationship between inflammation and the disorder of ECM and adhesion molecules.

Several limitations of this study need to be mentioned. First, we detected extensive changes in the expression levels of many inflammatory factors, ECM molecules, and adhesion molecules, but we did not identify the factor(s) that would be critical to induce the functional impairment of bone marrow stem cells. Second, we only screened three pathways in this study, but diabetes may also induce changes in many other pathways that contribute to the functional impairment of bone marrow stem cells. Further *in vitro* and *in vivo* studies will be needed to elucidate how these changes affect the growth and function of bone marrow stem cells.

In summary, c-kit^+^ bone marrow stem cells from diabetic mice exhibited extensive enhancement of inflammatory factors and changes in the expression of ECM and adhesion molecules, which might contribute to the decrease in the number of bone marrow stem cells and the impairment of their function for endothelial differentiation. The identification of key factor(s) in future studies will enable us to find novel approaches to prevent or reverse the diabetes-related functional impairment of stem cells.
